# Risk prediction for <1 cm lateral lymph node metastasis in papillary thyroid microcarcinoma

**DOI:** 10.3389/fendo.2023.1235354

**Published:** 2023-11-13

**Authors:** Chengxin Zhang, Siqi Fu, He Liu, Shuai Xue

**Affiliations:** ^1^ Business College, Guilin University Of Electronic Technology, Guilin, Guangxi, China; ^2^ General Surgery Center, Department of Thyroid Surgery, The 1st Hospital of Jilin University, Changchun, Jilin, China

**Keywords:** risk factor, predictive model, lateral lymph node metastasis, papillary thyroid microcarcinoma, lateral lymph node dissection

## Abstract

**Background:**

Because the diameter of the suspicious lymph nodes is less than 1 cm and adjacent to important structures in the neck, the diagnosis of small LLNM is important but difficult without the help of fine needle aspiration (FNA). There are no relevant reports of risk factors that predict the risk of suspicious <1 cm LLNM.

**Methods:**

A total of 159 PTMC patients with suspicious <1 cm LLNM were included in the study. Multivariate logistic regression analysis was used to identify ultrasound independent predictors of LLNM. A predictive model was developed according to multivariate logistic regression and evaluated by Hosmer-Lemeshow fit test.

**Results:**

Age ≤ 38 years old, the largest PTMC was located in the upper part, and the presence of liquefaction or microcalcification in suspicious lymph nodes were independent risk factors for LLNM (univariate analysis P = 0.00, 0.00, 0.00; multivariate analysis P = 0.00, 0.02, 0.00. OR = 4.66 [CI: 1.78-12.21], 3.04 [CI: 1.24-7.46], 6.39 [CI: 1.85-22.00]). The predictive model for the diagnosis of suspicious <1 cm lymph nodes was established as: P = e^x^/(1 + e^x^). X = -1.29 + (1.11 × whether the largest tumor is located in the upper part) + (1.54 × whether the age is ≤ 38 years) + (1.85 × whether the suspicious lymph nodes have liquefaction/microcalcification). The Hosmer-Lemeshow fit test was used to test the predicted ability, and it found that the predictive model had a good fit and prediction accuracy (X^2 = ^6.214, P = 0.623 > 0.05). Chi squared trend analysis showed that the increase in the number of risk factors gradually increased the malignancy possibility of suspicious <1 cm lymph nodes (chi squared trend test, P = 0.00).

**Conclusions:**

Age ≤ 38 years old, the largest PTMC located in the upper part, and the presence of liquefaction or microcalcification in suspicious lymph nodes were independent risk factors for suspicious <1 cm LLNM in PTMC patients. Our result show that it is feasible to evaluate the malignant possibility of these lymph nodes using the number of risk factors.

## 1 Introduction

The worldwide increasing incidence of thyroid cancer is primarily attributed to the rapid rise of papillary thyroid microcarcinoma (PTMC) (1). Most patients with PTMC were diagnosed using routine physical examination and had no clinical symptoms. The development and popularization of high-frequency ultrasound (US) and fine needle aspiration (FNA) in recent years have increased the diagnosis of PTMC (2, 3). Most PTMC are low-risk with a 1-5% of recurrence rate and a 0.3% 10-year mortality rate. Therefore, the use of a more conservative treatment strategy for low-risk PTMC is the current consensus. However, increasing studies reported that lateral lymph node metastasis (LLNM) was associated with locoregional recurrence, distant metastases and worse prognosis for PTMC (4, 5). The revised American Thyroid Association (ATA) guidelines in 2015 also considered that approximately 20% of PTC patients with LLNM would have structural recurrence in the future (6).

Approximately 20-50% of differentiated thyroid cancer patients found LLNM before surgery. The incidence of LLNM is less than 10% in PTMC. However, LLNM is a risk factor of high recurrence rate and poor prognosis in PTMC patients. Thyroid cancer guidelines suggest that only LLNM with definite preoperative or intraoperative diagnosis should be treated with therapeutic lateral lymph node dissection (LLND). Therefore, the diagnosis of LLNM is particularly important. Common preoperative examinations include US, Computed Tomography (CT), FNA and thyroglobulin (TG)-FNA. ATA guidelines recommend FNA only for ≥1 cm suspicious lateral lymph nodes (6). However, the diameter of suspicious lateral lymph nodes from many PTMC patients are less than 1 cm in the clinic. Because the diameter of the suspicious lymph nodes is small and adjacent to important structures of the neck, it is very difficult to diagnose using preoperative FNA. The diagnosis of small lymph nodes is only made from frozen sections during surgery. However, the frozen section diagnosis of lymph nodes during surgery may produce false-positive and false-negative results (7, 8). If it is misdiagnosed as negative, the patients must undergo re-operation after the corrected pathological diagnosis, which causes physical and psychological burden for the patients. If it is misdiagnosed as positive, the patient receives LLND, which may lead to more complications. Therefore, whether there are risk factors that predict the risk of suspicious <1 cm LLNM is not known.

We included patients who received LLND because of suspicious <1 cm lymph nodes, analyzed the clinical data and US characteristics, established a predictive diagnosis model, and evaluated its diagnostic ability. The present study improved the diagnosis of suspicious <1 cm lymph nodes in the lateral neck.

## 2 Materials and methods

### 2.1 Patient selection

The data of PTMC patients who underwent thyroid surgery in the First Hospital of Jilin University from January 2009 to July 2019 were analyzed retrospectively. The inclusion criteria of patients were complete information of patients in the hospital database, PTMC confirmed in postoperative pathology, suspicious <1 cm LLNM indicated on preoperative US, and suspicious lymph nodes were resected and confirmed by pathology. The exclusion criteria were age < 18 years old, neck radiotherapy history, and previous thyroid surgery history. A total of 159 PTMC patients with suspicious <1 cm LLNM were included in the study.

### 2.2 Diagnosis and treatment

Most patients with PTMC were diagnosed using US, which was performed by a trained radiologist, and re-evaluated by surgeons for thyroid tumors and cervical lymph nodes before surgery. It was suggested that FNA be performed preoperatively for the diagnosis of suspicious thyroid nodules. The ultrasonic manifestations of malignant lymph nodes are microcalcification, liquefaction, peripheral blood flow signal, hyperechoic, and round. If the largest diameter of suspicious lymph nodes in the neck was greater than 1.0 cm, then FNA was recommended. If the maximum diameter of the suspicious lymph nodes in the neck was smaller than 1.0 cm, then selective LLND was performed during the surgery, and frozen sectioning was performed to determine whether the suspicious lymph nodes were metastatic and whether modified radical LLND (at least II - IV area) should be performed.

### 2.3 Ultrasonic examination

Experienced US doctors in our department performed US examinations of thyroid nodules and lateral cervical lymph nodes before surgery. Thyroid nodules were primarily evaluated for its location, size, echo, texture, boundary, shape, blood flow and other characteristics. Most studies reported that the location of PTMC was a risk factor for LLNM, and we recorded the location of PTMC in detail. The size, location, shape, echo, calcification, blood flow and other characteristics of the lateral cervical lymph nodes were evaluated. We recorded this related information. Before the surgery, the clinician checked the accuracy of the US information again. If there was any inconformity, an experienced radiologist and surgeon were consulted.

### 2.4 Statistical analysis

Nominal variables are described as frequency and proportion, and continuous variables are described as means and standard deviation. To determine differences between specific variable groups, Pearson’s chi squared test was used for nominal variables, and Mann-Whitney U test was used for continuous variables. The risk factors were determined via univariate analysis and multivariate logistic regression analysis. The optimal cutoff value of continuous variable was determined using a receiver operating characteristic curve (ROC) and transformed into binary variables to better explain the results of risk factors. The prediction model of LLNM was calculated using the β coefficient provided by the multivariate logistic regression analysis. The ROC curve was used to evaluate the predictive ability of the predictive model. The Hosmer-Lemeshow fit test was used to test the predictive ability compared to the pathological results. Nonnormally distributed continuous variables were compared using the Kruskal Wallis test. The chi squared trend test was used to evaluate the correlation between the number of risk factors and the malignancy possibility of lateral cervical lymph nodes. P value < 0.05 was considered statistically significant. The 22nd edition SPSS software (SPSS Inc, Chicago, IL, USA) was used for all statistical analyses.

## 3 Results

### 3.1 General clinicopathological characteristics

**Table 1** summarizes the general clinicopathological characteristics of 159 PTMC patients with suspicious <1 cm lateral lymph nodes. One hundred and nineteen patients were female (74.8%) with an average age of 42.0 years. Most of the patients had the largest tumor in the upper part (57.9%). The shape of suspicious lymph nodes in the lateral neck of 49 patients (30.8%) was round or irregular. In 130 cases (81.8%) of the suspicious small lateral lymph nodes, no clear hilar structure was found. In 70 cases (44.0%), the suspicious lymph nodes were liquefied or microcalcified. Preoperative US diagnosis was correct in 121 cases, and the correct rate was 76.1%.

**Table 1:** Clinicopathological features of PTMC with suspicious <1cm lateral lymph nodes.

Variables	N=159 (%)
Sex	
Female	119 (74.8)
Male	40 (25.2)
Age, years	42.0±10.9
Location of largest tumor	
Upper	92 (57.9)
Non-upper	67 (42.1)
Shape	
Circle or irregular	49 (30.8)
Oval	110 (69.2)
Disappearance of lymphatic hilum	
Yes	130 (81.8)
No	29 (18.2)
Liquefaction or microcalfication	
Yes	70 (44.0)
No	89 (56.0)
Correct US diagnosis	
Yes	121 (76.1)
No	38 (23.9)

Categorical variables are presented as number (%, percentage). Continuous variables are presented as the average±standard deviation. PTMC, papillary thyroid microcarcinoma; US, ultrasound.

### 3.2 Univariate and multivariate analyses of risk factors

To examine the risk factors of LLNM in PTMC patients with suspicious <1 cm lateral lymph nodes, binary multivariate logistic regression analysis was performed. The continuous variable of age was converted into nominal variables using the cutoff value calculated in the ROC analysis (**Table 2**). As shown in **Tables 3** and **4**, univariate and multivariate analyses showed that age ≤ 38 years old, the largest PTMC located in the upper part, the presence of liquefaction or microcalcification in suspicious lymph nodes were independent risk factors for LLNM (univariate analysis P = 0.00, 0.00, 0.00; multivariate analysis P = 0.00, 0.02, 0.00. OR = 4.66 [CI: 1.78-12.21], 3.04 [CI: 1.24-7.46], 6.39 [CI: 1.85-22.00]).

**Table 2:** ROC analysis for optimal cutoff of continuous variables.

Variables	AUC (95% CI)	P value	Youden Index	Cutoff
Age	0.70 (0.63-0.77)	0.00	0.33	≤38

ROC, receiver operating characteristic curve; AUC, area under curve.

**Table 3:** Univariate analysis for risk factors of suspicious <1cm lateral lymph nodes in PTMC.

Variables	LLNM (N=159)		Univariate analysis
	Present, n=121	Absent, n=38	*P*
Sex			
Female	87 (71.9)	32 (84.2)	
Male	34 (28.1)	6 (15.8)	0.41
Age			
≤38 yr	65 (53.7)	8 (21.1)	
>38yr	56 (46.3)	30 (78.9)	0.00
Location of largest tumor			
Upper	79 (65.3)	13 (34.2)	
Non-upper	42 (34.7)	25 (65.8)	0.00
Shape			
Circle or irregular	46 (38.0)	3 (7.9)	
Oval	75 (62.0)	35 (92.1)	0.00
Disappearance of lymphatic hilum			
Yes	105 (86.8)	25 (65.8)	
No	16 (13.2)	13 (34.2)	0.00
Liquefaction or microcalfication			
Yes	66 (41.5)	4 (10.5)	
No	55 (58.5)	34 (89.5)	0.00

PTMC, papillary thyroid microcarcinoma; OR, odd ratio; LLNM, lateral lymph node metastasis.

**Table 4:** Multivariate analysis for risk factors of suspicious <1cm lateral lymph nodes in PTMC.

Variables	Multivariate analysis		
	*β*	OR (95% CI)	*P*
Sex			
Female		1 (reference)	
Male	0.64	1.89 (0.72-4.96)	0.20
Age			
≤38 yr		4.66 (1.78-12.21)	
>38yr	1.54	1 (reference)	0.00
Location of largest tumor			
Upper		3.04 (1.24-7.46)	
Non-upper	1.11	1 (reference)	0.02
Shape			
Circle or irregular		3.5 (0.57-22.03)	
Oval	0.87	1 (reference)	0.23
Disappearance of lymphatic hilum			
Yes		1.81 (0.65-5.03)	
No	0.59	1 (reference)	0.25
Liquefaction or microcalfication			
Yes		6.39 (1.85-22.00)	
No	1.85	1 (reference)	0.00
Constant	-1.29	1 (reference)	0.02

### 3.3 Establishment and evaluation of prediction model

**Figure 1:**
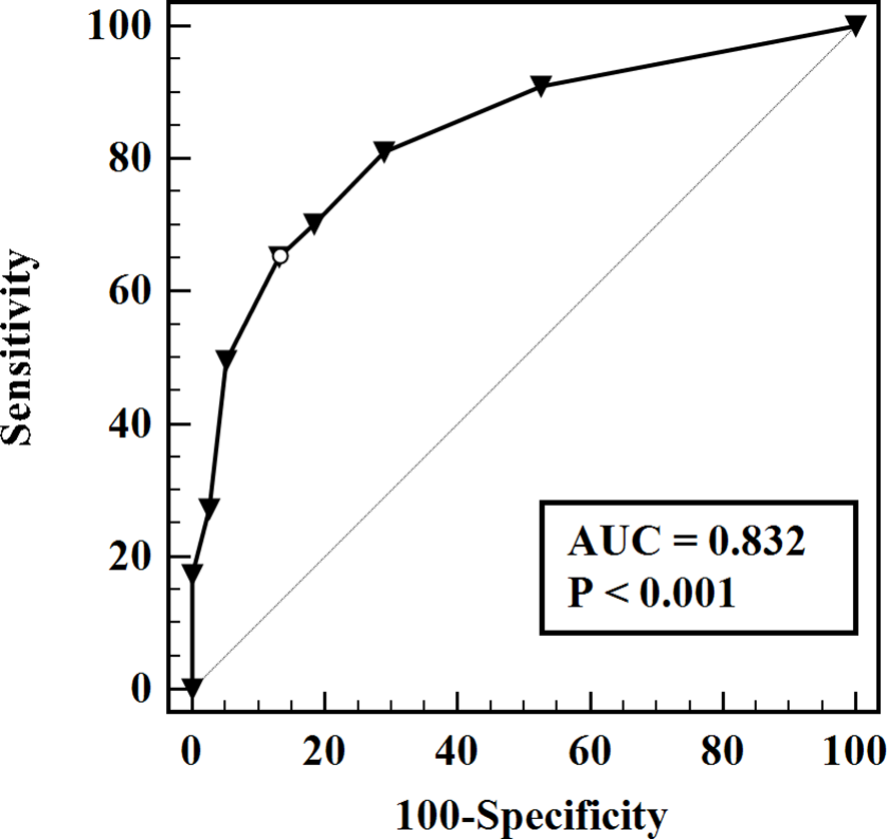
ROC evaluation for the prediction model.

The predictive model for the diagnosis of suspicious <1 cm lymph nodes was established as: P = e^x^/(1 + e^x^). X = -1.29 + (1.11 × whether the largest tumor is located in the upper part) + (1.54 × whether the age is ≤ 38 years) + (1.85 × whether the suspicious lymph nodes have liquefaction/microcalcification). P is the probability of malignant prediction, e is the natural logarithm, yes = 1, and no = 0. This group of data established the prediction model and calculated the value of malignant probability. An ROC curve was drawn according to the pathological results, as shown in **Figure 1**. The area under the curve was 0.832 ± 0.03, and the 95% confidence interval was 0.77-0.89. When the best cross-section value was 0.64, the sensitivity was 65.29%, and the specificity was 86.84%. The Hosmer-Lemeshow fit test was used to test the predictive ability and found that the predictive model had a good fit and prediction accuracy (X^2 = ^6.214, P = 0.623 > 0.05).

### 3.4 Relationship between the number of risk factors and the probability of malignancy prediction

**Figure 2:**
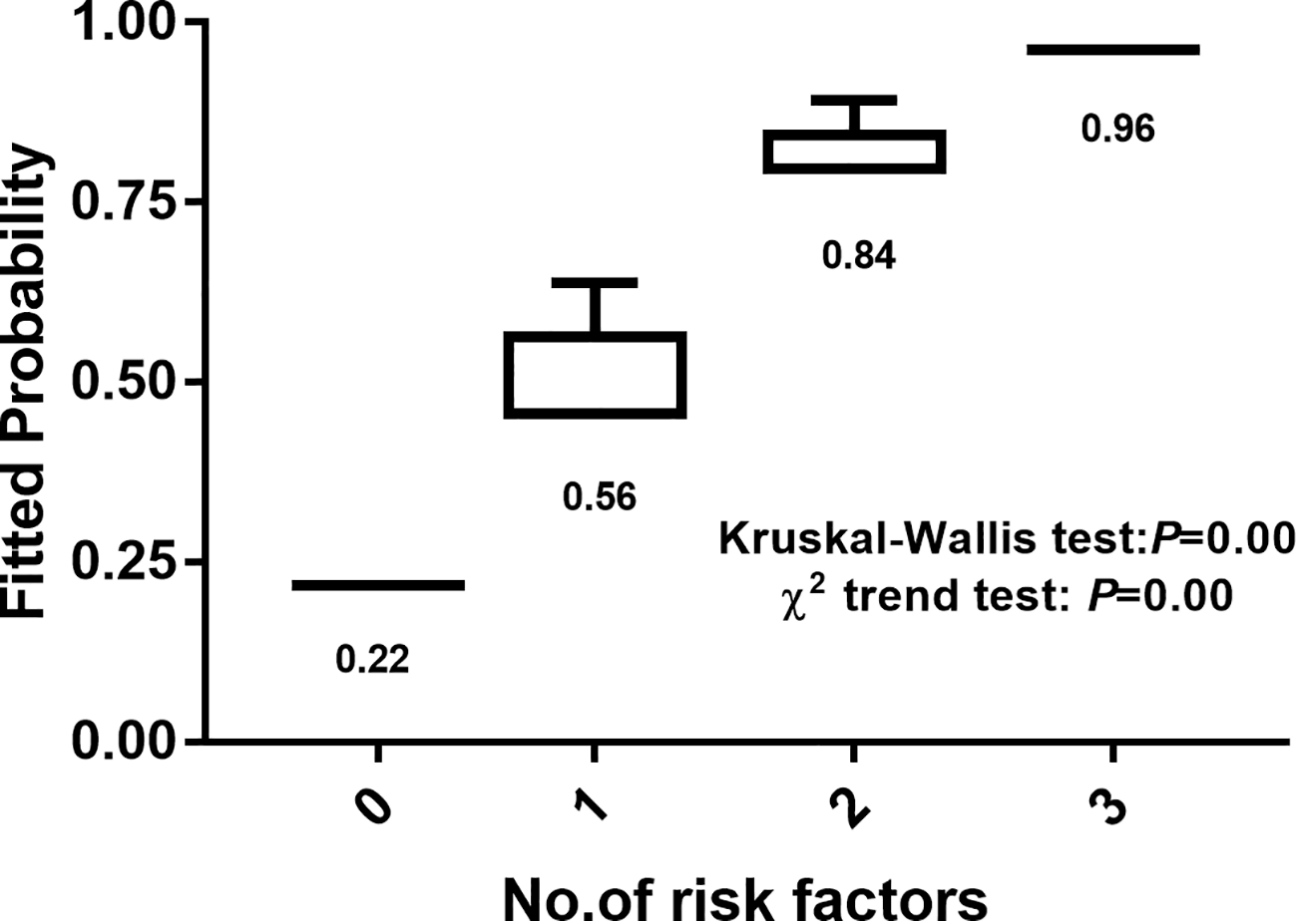
Relationship between number of risk factors and malignant prediction probability.

The number of risk factors was used as the abscissa, and the ordinate and box chart were created according to the malignant prediction probability calculated by the prediction model. There was a significant difference in malignant possibility between subgroups with different numbers of risk factors (Kruskal Wallis test, P = 0.00). The chi squared trend analysis showed that the malignancy possibility of suspicious <1 cm lymph nodes increased gradually with the increase of the number of risk factors (chi squared trend test, P = 0.00) (**Figure 2**).

## 4 Discussion

The diagnosis of suspicious <1 cm lateral lymph nodes is always difficult in clinical work. Because of the small size of the suspicious lymph nodes and adjacency to the important vessels, FNA before surgery is very difficult. The present study showed that 76.1% of the suspicious <1 cm lateral lymph nodes were diagnosed. However, 23.9% of the PTMC patients received radical LLND due to misdiagnosis, which caused physical and mental injury to these patients. Therefore, we determined some risk factors and established a prediction model to improve the accuracy of the diagnosis of suspicious <1 cm lateral lymph nodes.

Univariate and multivariate analyses found that age ≤ 38 years old, the largest tumor located in the upper part, and the presence of liquefaction or microcalcification in the suspicious lymph nodes were risk factors for suspicious <1 cm lateral lymph nodes. A large number of studies showed that age was a risk factor of LLNM in PTMC patients (9, 10). One active surveillance cohort study also found that young patients were more likely to have lymph node metastasis. Among ≤20 year-old PTMC patients under active surveillance, the possibility of LLNM during observation was as high as 11% (11). The specific mechanism of age-dependent tumorigenesis and development was not clear. The monitoring effect of the immune system on tumors changes with age, which may be the reason for the difference between young and old cancer patients (12, 13). Tumors located in the upper part were also closely related to LLNM of PTMC, especially with skip metastasis of PTMC (14–18). The reason may be that the lymph in the upper part of the thyroid gland flows into the lateral neck alone. Tumor in the upper part may not pass through the central neck region, and the tumor cells may be directly metastasized to the lateral neck, which may explain why thyroid cancer patients with tumors in the upper part have more LLNM (16). There are many malignant features of lymph nodes, such as round, irregular shape, high echo, uneven internal echo, unclear structure of lymphatics, liquefaction or microcalcification, and peripheral blood flow signal. Among these features, liquefaction and microcalcification have the highest specificity for the diagnosis of LLNM, which reaches 93-100% (6, 19, 20). Our study also found that liquefaction or microcalcification of the suspicious <1 cm lateral lymph nodes was very important for the diagnosis of LLNM.

There was no prediction model or grading system for the diagnosis of lateral cervical lymph nodes primarily due to the following reasons. 1) The sensitivity and specificity of US in the diagnosis of LLNM are high, at 62% - 100% and 68% - 98% respectively. 2) Preoperative CT combined with US further improved the diagnosis rate of metastatic lymph nodes in the lateral neck. 3) FNA may be used to directly diagnose a few suspicious lymph nodes. Therefore, many researchers believed that it was not necessary to risk-stratify LLNM. However, for suspicious <1 cm lateral lymph nodes, the diagnostic rate of US and CT is reduced. Because the lymph nodes are small and adjacent to important structures in the neck, FNA also has higher technical requirements for doctors. Therefore, it is very necessary to determine preoperative risk factors and examine a prediction model for suspicious <1 cm lateral lymph nodes.

Multivariate logistic regression analysis examined the prediction equation of malignant probability and verified its prediction efficiency. We found that this prediction model reliably predicted the benign and malignancy of suspicious <1 cm lateral lymph nodes. We also found that the number of risk factors positively correlated with the predicted malignant probability (chi squared trend test p = 0.00). Compared with the US grading system of thyroid nodules, the risk factors included were only the US characteristics of thyroid nodules. However, the prediction equation proposed in this experiment included a patient factor (age ≤ 38 years), a thyroid tumor factor (located in the upper part) and a US factor (liquefaction or microcalcification) of suspicious lymph nodes. Whether there is LLNM in PTMC patients is closely related to the predisposing factors of patients and the characteristics of thyroid cancer. Therefore, the incorporation of these factors may improve the prediction efficiency of the prediction model for suspicious lateral neck lymph nodes.

However, there are some limitations in this study. First, the number of patients included is too small, and there may be some bias in the multivariate analysis. The inclusion of more cases would improve the stability of the prediction model. Second, the prediction model obtained using calculation should be verified in another independent sample. However, due to the small number of PTMC with suspicious < 1 cm lymph nodes in the lateral neck, it was impossible to provide an independent case group to verify the correctness of the model. Finally, there is no strong evidence that <1 cm LLNM is closely related to a worse prognosis of PTMC patients, and whether the LLND for these lymph nodes less than 1 cm is beneficial to patients require verification in prospective randomized controlled trials.

## 5 Conclusion

Age ≤ 38 years old (18-38 years), the largest PTMC located in the upper part, and the presence of liquefaction or microcalcification in suspicious lymph nodes were independent risk factors for suspicious <1 cm LLNM in PTMC patients. The predictive model had better sensitivity and specificity for the diagnosis of suspicious <1 cm LLNM, and the diagnostic accuracy was high. The increase in the number of risk factors gradually increased the malignant possibility of the suspicious <1 cm lateral lymph nodes. It is feasible to evaluate the malignant possibility of the lymph nodes using the number of risk factors. However, independent case groups are needed to verify the accuracy and stability of the prediction model.

## Data availability statement

The raw data supporting the conclusions of this article will be made available by the authors, without undue reservation.

## Ethics statement

This study was approved by the Institutional Ethics Committee of First Hospital of Jilin University. Informed consent were gave to all these patients in the study.

## Author contributions

All authors contributed to the article and approved the submitted version.
